# Efficient Biotransformation of Zearalenone in Acidic Food Matrices by Alkaline Enzyme–Inorganic Hybrid Nanoflower

**DOI:** 10.3390/toxins18050229

**Published:** 2026-05-13

**Authors:** Ping Ding, Wenchao Liao, Chenyu Chen, Xincheng Chen, Chengfei Wang, Xiaoyang Li

**Affiliations:** 1 State Key Laboratory of Food Science and Resources, School of Food Science and Technology, Nanchang University, Nanchang 330047, China; bjyx20221109@163.com (P.D.); liaowenchao0214@163.com (W.L.); chenxc@email.ncu.edu.cn (X.C.); 2 Baichuan Biotechnology Co., Ltd., Wenzhou 325600, China; chency@baichuanfood.com; 3 Jiangsu Aomai Bio-Technology Co., Ltd., Nanjing 211225, China; chfwang@aomai-bio.com; 4 State Key Laboratory of Green Biomanufacturing, College of Life Science and Technology, Beijing University of Chemical Technology, Beijing 100029, China; 5 Beijing Synthetic Bio-Manufacturing Technology Innovation Center, Beijing 102209, China

**Keywords:** mycotoxins removal, zearalenone, biotransformation, zearalenone lactonase, enzyme immobilization

## Abstract

Zearalenone (ZEN) is a pervasive mycotoxin contaminating global food and feed. While enzymatic degradation offers a promising, specific, and eco-friendly strategy for mycotoxin mitigation, the biotransformation of ZEN within acidic food matrices remains challenging due to the intrinsically low activity of zearalenone lactonase (ZENG). In this work, we synthesized a ZENG–hydroxyapatite (Ca_10_(PO_4_)_6_(OH)_2_) hybrid nanoflower (CaNF) via biomineralization under alkaline conditions. Compared to free ZENG, the as-prepared biohybrid nanoflower exhibited markedly enhanced acid tolerance and catalytic activity, achieving a 12-fold increase in ZEN degradation efficiency at pH 5.0. Furthermore, the biohybrid nanoflower demonstrated robust performance in various acidic food matrices, including corn juice, wort, beer, and corn steep liquor. This study presents a powerful enzymatic tool for the efficient biotransformation of ZEN in acidic food-related systems.

## 1. Introduction

Zearalenone (ZEN), a mycotoxin produced by *Fusarium* species, is a pervasive contaminant in food and feed, particularly affecting maize, wheat, and their by-products [1,2,3,4,5,6]. ZEN poses significant toxicological risks due to its diverse adverse biological effects [1,2,3]. Conventional remediation strategies, such as physical adsorption and chemical treatments, are often hindered by limitations including high reagent consumption, potential secondary pollution, and the requirement for specialized equipment [7,8]. Consequently, the development of green, efficient, and environmentally sustainable strategies for ZEN removal is of paramount importance.

Enzymes, owing to their high substrate specificity, mild operating conditions, and environmental compatibility, have emerged as promising biocatalysts in food processing [9,10,11,12,13]. Enzymes that are used for ZEN degradation include lactonase [14,15], peroxidase [16,17], and laccase [18]. While peroxidases suffer from unclear catalytic mechanisms and laccases often require redox mediators, zearalenone-degrading lactonase (ZENG) stands out as a promising candidate. ZENG catalyzes the hydrolysis of the ester bond within the lactone ring of ZEN, effectively reducing its toxicity [19,20]. However, the practical application of ZENG is severely restricted by its poor stability and activity under acidic conditions [21]. This limitation is particularly critical given that many fermentation-related food matrices, such as corn juice, wort, and beer, are inherently acidic [22,23]. Therefore, enhancing the catalytic performance of ZENG in acidic media is essential for achieving efficient ZEN biotransformation in real-world food systems.

Enzyme catalytic efficiency is highly sensitive to pH because the protonation states of amino acid residues within the active site critically govern substrate binding and catalysis [24,25]. Consequently, modulating the enzyme microenvironment has emerged as a robust strategy to enhance enzymatic pH tolerance [26]. Conventional strategies encompass enzyme immobilization [27], covalent surface modification (e.g., grafting amino or carboxyl groups) [28], and the engineering of endogenous or biomimetic buffering microenvironments [29]. For instance, Shi et al. developed Fe-doped ZIF-8 encapsulating glucose oxidase (Fe-ZIF-8@GOx), where the carrier served as both a support and a protective shell, thereby improving catalytic performance over a broad pH range [30].

To enable efficient ZENG catalysis under acidic food matrices, we engineered a ZENG–hydroxyapatite (Ca_10_(PO_4_)_6_(OH)_2_) hybrid nanoflower (CaNF) via a biomineralization strategy. Owing to the abundant hydroxyl groups in hydroxyapatite, this matrix was hypothesized to create a more favorable microenvironment for the enzyme under acidic conditions. We systematically evaluated the catalytic performance and acid tolerance of the biohybrid nanoflowers in acidic matrices. This study presents a promising strategy to improve the catalytic performance of ZENG for ZEN biotransformation in acidic food systems.

## 2. Results

### 2.1. Preparation and Characterization of ZENG-HAP Hybrid Nanoflower

**Figure 1:**
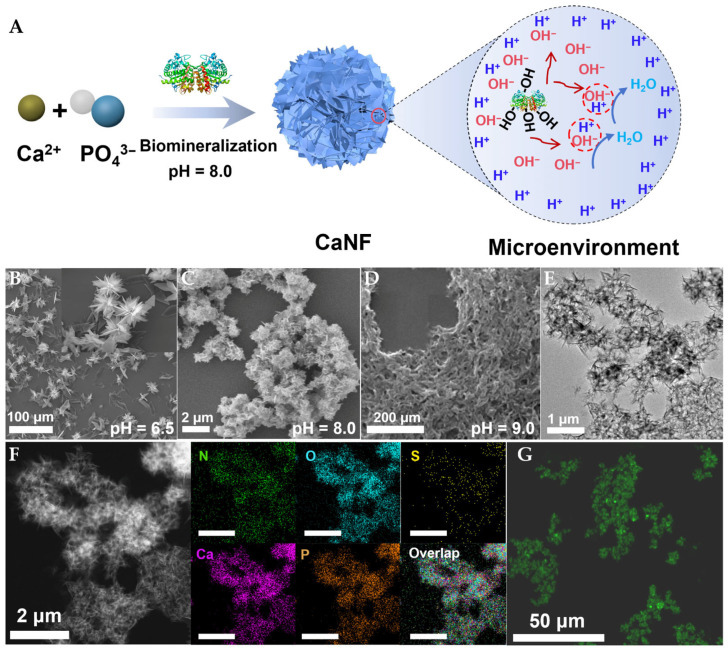
Synthesis and Characterization of CaNF. (**A**) Schematic diagram of CaNF synthesis under pH 8.0 conditions. (**B**) SEM image of CaNF-pH 6.5. (**C**) SEM image of CaNF-pH 8.0. (**D**) SEM image of CaNF-pH 9.0. (**E**) TEM image of CaNF-pH 8.0. (**F**) HAADF-STEM image of CaNF-pH 8.0 and the corresponding EDS mapping for N, O, S, Ca, and P. The scale bars in the figure are 2 μm. (**G**) LSCM image of CaNF-pH 8.0, with ZENG labeled with FITC (green).

The ZENG–hydroxyapatite (HAP) hybrid nanoflower (CaNF) was fabricated via a biomineralization strategy under alkaline conditions (Figure 1A). By adjusting the pH of the synthesis solution, the morphology of the nanoflower could be tuned. As a reference material, ZENG-CaHPO_4_ hybrid nanoflower was synthesized at pH 6.5. The scanning electron microscopy (SEM) images of CaNF synthesized under different pH conditions are shown in Figure 1B–D. At pH 6.5, the synthesized nanoflower exhibited a floral morphology with flaky petals (CaNF-pH 6.5) (Figure 1B). At pH 8.0, the size of CaNF significantly decreased, with an average size of 0.9 μm (CaNF-pH 8.0) (Figure 1C). At pH 9.0, the ZENG-HAP complex showed a network-like structure (CaNF-pH 9.0) (Figure 1D).

The transmission electron microscopy (TEM) and high-angle annular dark-field scanning transmission electron microscopy (HAADF-STEM) images of CaNF-pH 8.0 further confirmed the formation of nanoflower (Figure 1E,F). The elemental composition of CaNF was characterized by energy-dispersive X-ray spectroscopy (EDS) mapping. As shown in Figure 1F, CaNF contained N, O, S, Ca, and P elements. The N and S elements originated from ZENG, whereas the Ca and P elements were derived from HAP, confirming the construction of the ZENG-HAP complex. Laser scanning confocal microscopy (LSCM) was further used to characterize enzyme immobilization on the nanoflower. Green fluorescence was clearly observed in the LSCM image of CaNF prepared with FITC-labeled ZENG, indicating successful enzyme immobilization (Figure 1G).

**Figure 2:**
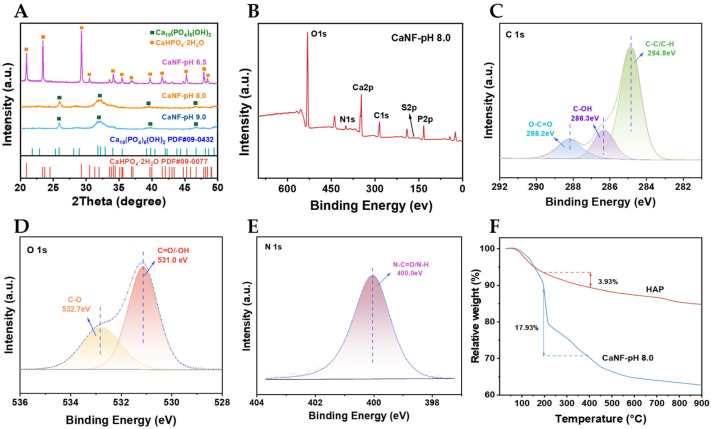
Structural characterization of CaNF. (**A**) XRD patterns of the CaNF synthesized at different pH levels. (**B**) XPS survey spectrum of CaNF-pH 8.0. (**C**) C 1s spectrum, (**D**) O 1s spectrum, and (**E**) N 1s spectrum of CaNF-pH 8.0. (**F**) Thermogravimetric analysis curves of CaNF-pH 8.0 and HAP.

The crystal structures of CaNF-pH 6.5, CaNF-pH 8.0, and CaNF-pH 9.0 were determined by X-ray diffraction (XRD), and the corresponding patterns are shown in Figure 2A. CaNF-pH 6.5 exhibited well-defined reflections matching CaHPO_4_·2H_2_O, whereas CaNF-pH 8.0 and CaNF-pH 9.0 matched well with Ca_10_(PO_4_)_6_(OH)_2_ (HAP). The molar ratio of Ca to P in CaNF-pH 8.0, determined by inductively coupled plasma-atomic emission spectrometry (ICP-AES), was 1.6, which is consistent with the stoichiometric ratio of HAP (Table S1).

The chemical state of CaNF-pH 8.0 was analyzed by X-ray photoelectron spectroscopy (XPS). The XPS survey spectrum (Figure 2B) showed the presence of C, O, N, P, S, and Ca. In the C 1s spectrum, the peaks at 284.8, 286.3, and 288.2 eV were attributed to C-C/C-H, C-OH, and O-C=O bonds, respectively (Figure 2C). In the O 1s spectrum, the peaks at 531.0 and 532.7 eV corresponded to the C=O bond of ZENG or the -OH group of HAP, and the C-O bond of ZENG, respectively (Figure 2D). In the N 1s spectrum, the peak at 400.0 eV was assigned to N-C=O and N-H bonds originating from ZENG (Figure 2E).

The protein weight percentage of CaNF-pH 8.0 was further analyzed by thermogravimetric analysis (TGA), with HAP used as the reference material. As shown in Figure 2F, the weight losses of CaNF-pH 8.0 and HAP between 200 and 400 °C were 17.93% and 3.93%, respectively, indicating the presence of organic components (protein) in the CaNF structure. The protein content was estimated to be approximately 14%, which is in general agreement with the BCA results. These results confirm the successful immobilization of ZENG, while the quantitative enzyme loading used for catalytic normalization was determined exclusively by the BCA assay. Fourier-transform infrared spectroscopy (FT-IR) was performed to further determine the molecular components of CaNF (Figure S1). Both CaNF-pH 6.5 and CaNF-pH 8.0 showed strong IR bands at 1039 and 558 cm^−1^, corresponding to the asymmetric and symmetric stretching vibrations of PO_4_^3−^. Meanwhile, the peak at 1654 cm^−1^ was attributed to the C=O stretching vibration of ZENG.

The immobilization efficiency of ZENG was quantitatively determined by the BCA assay based on the residual protein content in the supernatant after immobilization. As shown in Figure S2, the immobilization efficiency varied with initial ZENG concentration. When the ZENG concentration was 0.025 mg/mL, the immobilization efficiency was nearly 100%, while it decreased to 28.0% and 57.4% at 0.05 and 0.1 mg/mL, respectively. These results were further used to calculate the enzyme loading content of CaNF, which served as the basis for normalization of enzyme dosage in subsequent catalytic experiments and TON analysis.

### 2.2. Degradation of ZEN in Acidic Matrices Catalyzed by CaNF

**Figure 3:**
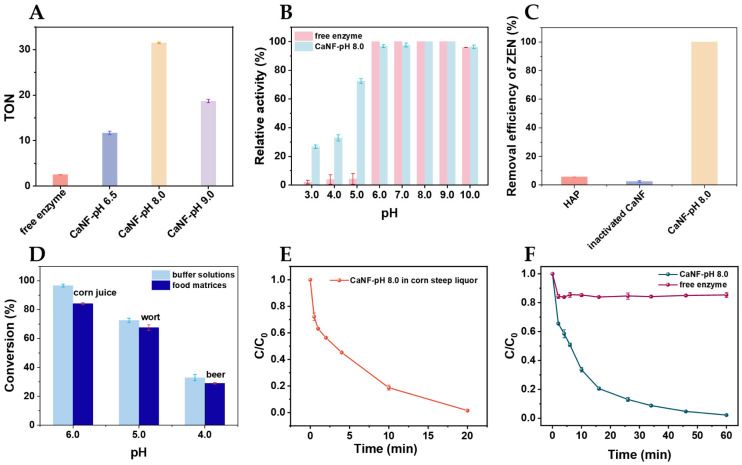
Degradation of ZEN catalyzed by CaNF. (**A**) Turnover number of free enzyme and CaNF toward ZEN at pH 5.0. (**B**) Relative activities of free ZENG and CaNF-pH 8.0 under different pH conditions. Equal amounts of enzyme were used in all catalytic systems. (**C**) Removal of ZEN by CaNF-pH 8.0. HAP and inactivated CaNF are used as the reference materials. Conditions: CaNF-pH 8.0 (40 μg, containing 4 μg ZENG), HAP (40 μg), inactivated CaNF (40 μg, containing 4 μg ZENG), ZEN (1000 μg/kg), and reaction time of 20 min. (**D**) Conversion of ZEN by CaNF-pH 8.0 in different matrices at different pH values. Conditions: CaNF-pH 8.0 (containing 4 μg ZENG), ZEN (1000 μg/kg), and reaction time of 20 min. (**E**) Catalytic performance of CaNF-pH 8.0 in corn steep liquor. C_0_ and C are the concentrations of ZEN before and after the reaction, respectively. Conditions: CaNF (40 μg, containing 4 μg ZENG), ZEN (800 μg/kg), and reaction time of 20 min. (**F**) Comparison of the catalytic performance of free enzyme and CaNF-pH 8.0. Equal amounts of enzyme were used in both catalytic systems. C_0_ and C are the concentrations of ZEN before and after the reaction, respectively.

The catalytic performance of CaNF for ZEN degradation was evaluated under acidic conditions. The turnover number (TON) of CaNF-pH 8.0 for ZEN degradation at pH 5.0 was 31.5, which was 12.6-, 2.7-, and 1.7-fold higher than those of the free enzyme, CaNF-pH 6.5, and CaNF-pH 9.0, respectively, under the same nominal enzyme dosage normalized based on the BCA-determined enzyme loading content, ensuring a fair comparison between free and immobilized enzyme systems (Figure 3A).

The amount of CaNF used in each reaction was adjusted according to the enzyme loading content, and the corresponding amount of free ZENG was used as a control. Detailed normalization information is provided in Table S2.

The activity of CaNF-pH 8.0 over a pH range of 3.0–10.0 was further determined. As shown in Figure 3B, both the free enzyme and CaNF exhibited high activity when the pH ranged from 6.0 to 10.0. When the pH was below 6.0, the activity of the free enzyme markedly decreased, whereas CaNF maintained relatively high activity in acidic media. At pH 3.0, 4.0, and 5.0, the relative activities of the free enzyme were 2.1%, 3.8%, and 4.4%, respectively. Under the same conditions, the corresponding relative activities of CaNF were 26.8%, 33.0%, and 72.6%, respectively. The temperature dependence of CaNF-pH 8.0 was also investigated. As shown in Figure S3, CaNF-pH 8.0 exhibited the highest catalytic activity at 40 °C, and the activity declined when the temperature was increased to 60 °C or decreased to 10 °C.

To distinguish enzymatic catalysis from non-enzymatic ZEN removal, the ZEN removal efficiency of CaNF-pH 8.0 was measured using HAP and inactivated CaNF as the reference materials. As shown in Figure 3C, the ZEN removal efficiencies of HAP and inactivated CaNF were 5.7% and 2.5%, respectively, whereas the ZEN removal efficiency reached 100% when using CaNF-pH 8.0 under the same conditions (pH 5.0).

The catalytic performance of CaNF was evaluated in several representative acidic food matrices, including corn juice, wort, beer, and corn steep liquor. The pH values of corn juice, wort, and beer were 6.0, 5.0, and 4.0, respectively. As shown in Figure 3D, the conversion of ZEN catalyzed by CaNF in corn juice, wort, and beer was 84.1%, 67.5%, and 33.0%, respectively. In corn steep liquor (pH 5.0), nearly 100% of ZEN was transformed within 20 min (Figure 3E). To further assess the influence of matrix conditions, the degradation efficiencies in corn juice, wort, and beer were compared with those obtained in buffer solutions at the corresponding pH values. The degradation efficiencies in corn juice and wort were close to those of the corresponding buffer controls, whereas a lower degradation efficiency was observed in beer than in the buffer system at pH 4.0. These results suggest that the catalytic performance of CaNF in real food matrices was affected by both pH and matrix environment.

The catalytic kinetic curves of CaNF-pH 8.0 and the free enzyme are shown in Figure 3F. Within 60 min, the degradation rates of ZEN catalyzed by CaNF-pH 8.0 and the free enzyme reached 97.8% and 14.6%, respectively. As shown in Table 1, the Km values for CaNF and free ZENG were 3.95 μM and 8.21 μM, respectively, whereas the Kcat values were 0.124 s^−1^ and 0.051 s^−1^, respectively. The corresponding Michaelis–Menten curves are shown in Figure S4. These results indicated that CaNF exhibited improved catalytic performance compared with the free enzyme.

### 2.3. Reusability and Storage Stability of CaNF

**Figure 4:**
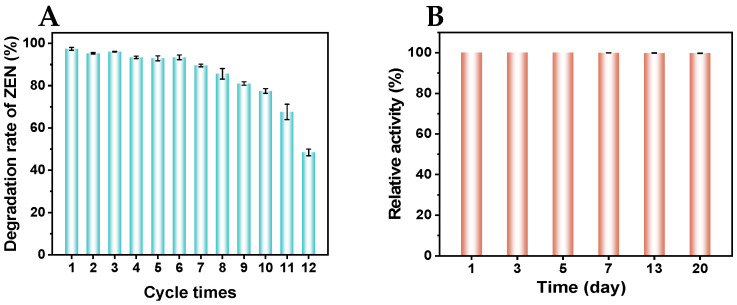
Reusability and storage stability of CaNF. (**A**) Degradation rate of ZEN in twelve cycles using CaNF as the catalyst. (**B**) The relative activity of CaNF-pH 8.0 at different storage time in degradation of ZEN.

CaNF could be readily separated by centrifugation after reaction and reused in subsequent cycles. As shown in Figure 4A, the immobilized enzyme maintained nearly 50% residual activity after twelve reuse cycles. The storage stability of CaNF-pH 8.0 was further evaluated by measuring its relative activity after storage for different periods. As shown in Figure 4B, CaNF retained nearly 100% residual activity after storage at 4 °C for 20 days.

### 2.4. Preliminary Evaluation of the HPLC Method for ZEN Determination in Selected Food Matrices

To provide a preliminary assessment of the proposed HPLC method in selected food matrices, single-level spike-recovery experiments were conducted in corn juice, wort, beer, and corn steep liquor. As shown in Table 2, at a spiking level of 1000 μg/kg, the recoveries of ZEN ranged from 87.98% to 97.63%, with RSD values ranging from 0.23% to 3.54%.

In addition, the calibration curve is shown in Figure S5, and the HPLC chromatogram of the ZEN standard is shown in Figure S6. ZEN was detected at a retention time of 7.3–7.6 min, and the peak area increased with increasing concentration. The LOD and LOQ of the HPLC method were 0.82 and 2.73 ng/mL, respectively, based on signal-to-noise ratios of 3 and 10 (Table S3).

### 2.5. COMSOL Simulation of Local Proton Distribution in ZENG-HAP Hybrid Nanoflowers Under Acidic Conditions

**Figure 5:**
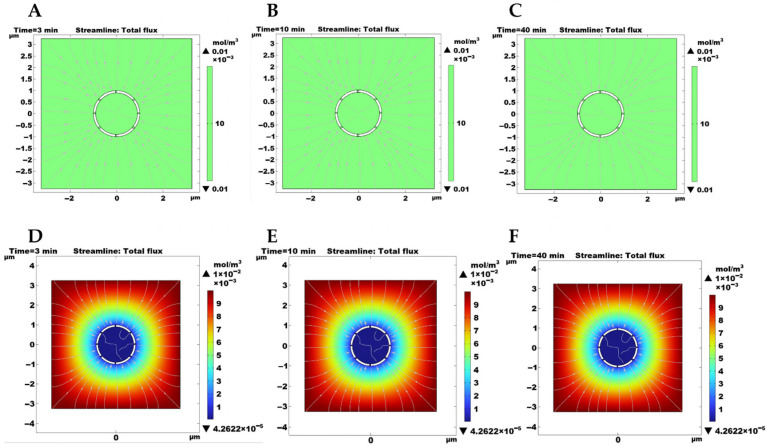
COMSOL simulation of the local proton distribution in ZENG-HAP hybrid nanoflowers under acidic conditions (pH 5.0). A non-HAP porous model with the same geometry was used as the reference. (**A**–**C**) Simulation of the non-HAP immobilized enzyme system at 3, 10, and 40 min. (**D**–**F**) Simulation of the ZENG-HAP hybrid nanoflowers system at 3, 10, and 40 min.

COMSOL simulations (version 5.6, COMSOL AB, Stockholm, Sweden) were performed to further investigate the effect of hydroxyapatite (HAP) on the enzymatic activity under acidic conditions. The model of the biohybrid nanoflower is constructed as a HAP porous material. The model of the conventional immobilized enzyme is constructed as a non-HAP porous material. The proton distributions in these models under acidic conditions (pH 5.0) were simulated using COMSOL. In the non-HAP immobilized enzyme system, hydrogen ions diffused uniformly throughout the reaction medium, showing that the hydrogen ion concentrations inside and outside the material are equal (Figure 5A–C). In contrast, in the enzyme-HAP hybrid materials, the hydrogen ion concentration within the material is significantly lower than that outside the material, indicating that HAP could influence local proton distribution within the porous structure under acidic conditions, thereby potentially enhancing the enzyme’s activity (Figure 5D–F).

### 2.6. Identification and Quantification of ZEN Transformation Product

**Figure 6:**
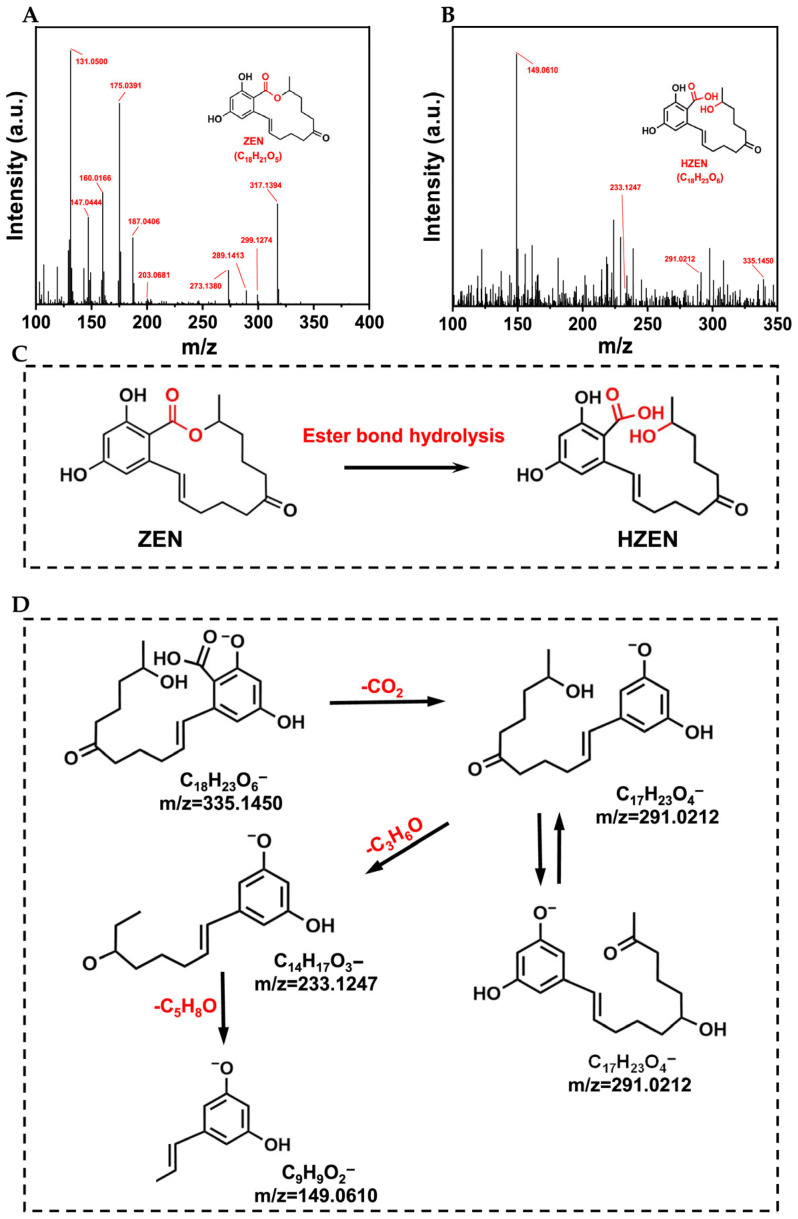
Identification of the ZEN transformation product catalyzed by CaNF. (**A**) MS spectrum of ZEN. (**B**) MS spectrum of HZEN. (**C**) Possible transformation pathway from ZEN to HZEN. (**D**) Possible dissociation pathway of HZEN.

The molecular structure of the ZEN transformation product was determined by ultrahigh-performance liquid chromatography coupled with quadrupole time-of-flight mass spectrometry (UPLC-Q-TOF/MS) in negative-ion mode. The transformation product was obtained and purified using preparative HPLC. The MS spectra of ZEN and its transformation product HZEN are shown in Figure 6A,B. The proposed transformation pathway of ZEN catalyzed by CaNF is presented in Figure 6C. The ester bond of ZEN (m/z = 317.1394) was cleaved, yielding the hydrolysis product HZEN (m/z = 335.1450). The molecular formula of the transformation product was predicted using MassHunter Qualitative Analysis software (Agilent Technologies, Santa Clara, CA, USA). Based on the fragment ions of the transformation product obtained by UPLC-Q-TOF/MS (Figures S8 and S9), the possible dissociation pathways of ZEN and HZEN were proposed (Figure S10 and Figure 6D).

The molar-balance between ZEN disappearance and HZEN formation was further analyzed. As shown in Table S4, from an initial ZEN concentration of 10.00 μM, 9.33 ± 0.13 μM ZEN was consumed and 8.99 ± 0.21 μM HZEN was formed. The formation of HZEN accounted for 96.39 ± 0.89% of the consumed ZEN on a molar basis. The overall molar recovery, calculated as the sum of remaining ZEN and formed HZEN relative to the initial ZEN, was 96.64 ± 0.78%. These results demonstrate that HZEN formation accounted for the majority of ZEN disappearance during CaNF-catalyzed ZEN transformation, with some minor experimental loss inherent to the system.

### 2.7. Cell Viability Assay

ZEN is known to exert toxic effects on the reproductive system and the liver [31,32]. The toxicological properties of ZEN and HZEN were further estimated in silico using the U.S. EPA ECOSAR (v1.0) and T.E.S.T. (v 4.2) programs. The predicted acute toxicity, chronic toxicity, developmental toxicity, mutagenicity, and bioaccumulation factor are summarized in Table S5. According to the ECOSAR results, HZEN showed higher acute and chronic toxicity thresholds for fish, daphnid, and green algae than ZEN, suggesting lower predicted aquatic toxicity. However, not all predicted hazards were eliminated, since developmental toxicity remained positive for both ZEN and HZEN in the T.E.S.T. prediction.

**Figure 7:**
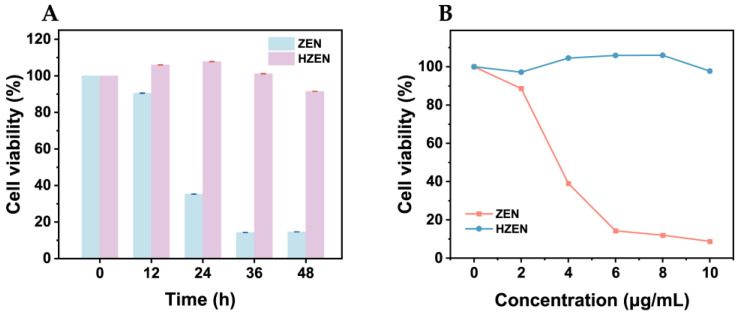
Cytotoxicity of ZEN and its transformation product HZEN in HepG2 cells. (**A**) Viability of human hepatoma HepG2 cells after exposure to ZEN or HZEN for different durations. (**B**) Viability of HepG2 cells after 48 h exposure to different concentrations of ZEN or HZEN.

The cytotoxicity of ZEN and its transformation product HZEN was evaluated using a cell viability assay in HepG2 cells. After exposure for 48 h at 6 μg/mL, the viability of human hepatoma HepG2 cells was 14.6% and 91.5% for ZEN and HZEN, respectively (Figure 7A). Figure 7B further shows the viability of HepG2 cells exposed to a series of concentrations of ZEN and HZEN. No significant decrease in cell viability in this hepatic cell model was observed at concentrations below 2 μg/mL for either group. When the concentration exceeded 2 μg/mL, the viability of ZEN-treated HepG2 cells gradually decreased with increasing concentration and dropped to less than 10% at 10 μg/mL, whereas HZEN showed only negligible effects on cell viability within the tested range (Figure 7B). These results indicate that HZEN exhibited reduced cytotoxicity than ZEN in this hepatic cell model, but this does not necessarily imply reduced endocrine or overall toxicological risk.

## 3. Discussion

### 3.1. ZENG-HAP Hybrid Nanoflower Boosts the ZEN Biotransformation Efficiency Under Acidic Conditions

The present study shows that alkaline biomineralization is a promising strategy for constructing a ZENG-HAP hybrid nanoflower (CaNF) with enhanced catalytic performance under acidic conditions [33,34,35]. Previous studies on enzyme-inorganic hybrid nanoflowers have demonstrated that the synthesis conditions, particularly pH, strongly influence the mineral phase, morphology, and enzyme incorporation efficiency of the resulting materials [33,35,36]. Consistent with this understanding, our results showed that varying the synthesis pH markedly altered both the crystal composition and morphology of the hybrid structures. Specifically, a pH of 8.0 yielded a HAP-based nanoflower structure characterized by relatively uniform morphology and clear evidence of successful ZENG incorporation. In contrast, a pH of 6.5 yielded a CaHPO_4_-based nanoflower structure, while a pH of 9.0 resulted in a network-like morphology of enzyme-HAP hybrid nanomaterials.

These differences in structure were closely associated with catalytic performance. Among the tested materials, CaNF-pH 8.0 exhibited the highest activity toward ZEN degradation under acidic conditions, suggesting that both the mineral phase and the structural organization of the carrier may have contributed to the observed catalytic differences.

The most significant finding of this study is that CaNF retained substantially higher catalytic activity than the free enzyme under acidic conditions, where the free enzyme was nearly inactive. Enzyme immobilization has long been recognized as an effective strategy for improving enzyme stability and reusability [26,37]. In this work, the results demonstrate that enzyme immobilization can not only stabilize enzyme structure, but also provide a more favorable catalytic environment for ZENG in acidic media. This is particularly important because acid tolerance is a prerequisite for the practical application of ZEN-degrading enzymes in food and feed systems [22,38].

Control experiments further demonstrated that neither HAP alone nor inactivated CaNF contributed appreciably to ZEN removal, indicating that the superior performance of CaNF mainly arose from retained enzymatic catalysis rather than adsorption or matrix effects of the composite. In addition, CaNF exhibited a lower Km value and a higher Kcat value than the free enzyme, indicating a higher apparent substrate affinity and faster catalytic turnover. Accordingly, the calculated Kcat/Km value of CaNF was markedly higher than that of free ZENG, reflecting an overall improvement in catalytic efficiency after immobilization. These kinetic results are consistent with the rapid ZEN degradation observed for CaNF and suggest that the immobilization strategy improved not only enzyme stability but also catalytic effectiveness under acidic conditions.

These simulation results indicate that HAP could influence the local proton distribution within the hybrid nanoflower under acidic conditions. In the non-HAP model, hydrogen ions were uniformly distributed throughout the medium, indicating that the enzyme would be directly exposed to a homogeneous acidic environment. In contrast, the HAP model maintained a relatively stable proton concentration around the immobilized enzyme, thereby potentially contributing to a more favorable local microenvironment for catalysis [33,39]. Although this mechanism still requires more direct experimental verification, the combined catalytic, kinetic, and simulation results suggest that microenvironment regulation may play a key role in the superior acidic performance of CaNF [26,37].

### 3.2. ZENG-HAP Hybrid Nanoflower Shows Promising Applications in Acidic Food Matrices

CaNF exhibited excellent efficiency in representative acidic food-related matrices such as corn juice, wort, beer, and corn steep liquor, highlighting its potential for practical ZEN biotransformation. Notably, corn steep liquor (pH 5.0) achieved nearly complete ZEN conversion, suggesting that matrix complexity alone does not dictate efficiency. Conversely, performance in beer (pH 4.0) was significantly reduced compared to its buffer control, indicating that the lower pH, potentially compounded by matrix-specific factors (e.g., sugars, polyphenols), imposes practical limits. These findings define an optimal operating window for CaNF around pH 5.0–6.0, where it effectively overcomes matrix interference.

The analytical results provide preliminary support for the application of the HPLC method to the acidic food matrices. UPLC-Q-TOF/MS analysis confirmed the formation of HZEN, suggesting that ZEN degradation was accompanied by enzymatic transformation rather than simple disappearance. The transformation product HZEN exhibited reduced cytotoxicity than ZEN in HepG2 cells under the tested conditions. Nevertheless, this result should be interpreted with caution, as the HepG2 assay reflects only general cytotoxicity in a hepatic cell model and does not evaluate estrogenic or endocrine activity, which is central to ZEN toxicity. In summary, these findings indicate that the current data support reduced cytotoxicity only, rather than overall detoxification or toxicological safety. Further studies addressing endocrine and developmental effects are therefore needed.

### 3.3. Limitations and Future Perspectives

Although differences in catalytic performance were observed depending on the matrix, particularly under strongly acidic conditions (e.g., beer at pH 4.0), the specific inhibitory components in complex food systems have not been systematically identified or studied. More systematic investigation of matrix effects, including component-specific inhibition, interactions with food constituents (e.g., sugars, proteins, and polyphenols), and long-term operational stability, will be needed to better define the practical application limits and operational window. Based on these consideration, future studies should focus on further validation across a broader range of food matrices and under more diverse and prolonged operational conditions, including long-term stability testing. In addition, although appropriate buffer systems were selected for different pH intervals, continuous monitoring of pH drift during the reaction was not performed. Therefore, minor pH changes during the reaction cannot be fully excluded. Although the HPLC method showed acceptable preliminary analytical performance, more rigorous analytical validation, including matrix-matched calibration, matrix effects, extraction efficiency, dilution integrity, and multi-level recovery would be required before its routine quantitative application in complex food matrices.

## 4. Conclusions

This study demonstrates a facile strategy to overcome acid-induced inactivation of ZENG for practical decontamination of ZEN in acidic food matrices. By constructing a ZENG-HAP hybrid nanoflower via biomineralization at pH 8.0, we proposed that the hydroxyl-rich surface of hydroxyapatite may contribute to a more favorable microenvironment around the enzyme. The resulting CaNF exhibited markedly enhanced catalytic performance under acidic conditions (pH 3.0–5.0), achieving a 12-fold higher ZEN degradation efficiency than free ZENG at pH 5.0. Importantly, CaNF maintained excellent activity in real acidic food systems including corn juice, wort, beer, and corn steep liquor, where native ZENG showed no detectable activity, highlighting strong potential for application in food processing.

## 5. Materials and Methods

### 5.1. Chemicals and Reagents

Zearalenone lactonase (ZENG, 1500 U/mg; one unit of enzyme activity was defined as the amount of enzyme required to degrade 1 μmol of ZEN per min under the optimal pH condition at 30 °C) and corn steep liquor were provided by Jiangsu Aomai Biotechnology Co., Ltd. (Nanjing, China). Zearalenone (ZEN) standard (purity > 99%) was purchased from Pribolab (Qingdao, China). Disodium hydrogen phosphate dodecahydrate (Na_2_HPO_4_·12H_2_O, AR, 99%), sodium dihydrogen phosphate dihydrate (NaH_2_PO_4_·2H_2_O, AR, 99%), citric acid (AR, 99%), sodium citrate dihydrate (AR, 99%), sodium carbonate (Na_2_CO_3_, AR, 99%), sodium bicarbonate (NaHCO_3_, AR, 99%), and anhydrous calcium chloride (CaCl_2_, AR, 99%) were purchased from Xilong Scientific Co., Ltd. (Guangzhou, China). Fluorescein 5 (6) -isothiocyanate (FITC) was obtained from Sigma-Aldrich (St. Louis, MO, USA). A BCA Protein Assay Kit was purchased from Beyotime Biotechnology Co., Ltd. (Shanghai, China). Acetonitrile and methanol (HPLC grade) were obtained from Tedia Company Inc. (Fairfield, OH, USA) and filtered through a 0.22 μm organic membrane before use. Formic acid (purity ≥ 98%) was purchased from Merck KGaA (Darmstadt, Germany) and filtered through a 0.22 μm organic membrane before use. Corn juice, wort, and beer were purchased from local commercial sources. All other chemicals and reagents were of analytical grade. Ultrapure water was used throughout the experiments.

### 5.2. Preparation of ZENG-HAP Nanoflowers

CaNF-pH 6.5, CaNF-pH 8.0, and CaNF-pH 9.0 were prepared using the same biomineralization procedure, with the pH of the phosphate buffer adjusted to 6.5, 8.0, or 9.0, respectively. Briefly, 100 mL of phosphate buffer (PB, 10 mM) was prepared, and the pH was adjusted to 6.5, 8.0, or 9.0. The prepared buffer solutions were equilibrated in a water bath at 30 °C before use. ZENG was then added to each buffer solution to a final concentration of 25 μg/mL, followed by the rapid addition of 44 mg of anhydrous CaCl_2_. The mixture was stirred at 350 rpm for 10 min and subsequently incubated at 25 °C for 12 h to allow nanoflower formation. After incubation, the suspension was centrifuged at 12,000 rpm for 5 min at 4 °C. The supernatant was discarded, and the precipitate was washed three times with ultrapure water. The resulting solid was dried under vacuum overnight to obtain the corresponding nanoflowers, namely, CaNF-pH 6.5, CaNF-pH 8.0, and CaNF-pH 9.0.

### 5.3. Determination of ZENG Loading

The immobilization efficiency of ZENG in CaNF was determined using a BCA protein assay kit (Beyotime Biotechnology Co., Ltd., Shanghai, China). After CaNF synthesis, the suspension was centrifuged to collect the supernatant. An aliquot of the supernatant was mixed with BCA working reagent and incubated at 37 °C for 30 min according to the manufacturer’s instructions. The absorbance at 562 nm was measured using a microplate reader. The concentration of unimmobilized ZENG in the supernatant was determined from a standard calibration curve, and the immobilization efficiency was calculated based on the difference between the initial amount of enzyme added and the amount remaining in the supernatant.

Immobilization efficiency (%) = (C_0_ − C_1_)/C_0_ × 100, (1)

where C_0_ is the initial concentration of ZENG added and C_1_ is the concentration of ZENG remaining in the supernatant after synthesis.

Immobilization efficiency was defined as the percentage of the initial enzyme amount that became immobilized onto the carrier, whereas enzyme loading content was defined as the mass percentage of immobilized enzyme in the final CaNF material.

For catalytic comparisons, the amount of CaNF used in each reaction was normalized based on the BCA-determined enzyme loading content, and the same nominal amount of free ZENG was used in the corresponding control experiments to ensure comparable enzyme input across different systems.

### 5.4. Degradation of ZEN in Buffer Solution Using CaNF

The ZEN degradation reaction was carried out in a water bath under magnetic stirring. To evaluate the effects of reaction conditions on ZEN degradation, different buffer systems, temperatures, and catalysts were investigated, including citric acid/sodium citrate buffer (pH 3.0–5.0), phosphate buffer (PB, pH 6.0–8.0), sodium carbonate/sodium bicarbonate buffer (pH 9.0–10.0), all buffers were prepared at 10 mM, reaction temperatures ranging from 10 to 60 °C, and different catalysts (free ZENG, CaNF-pH 6.5, CaNF-pH 8.0, and CaNF-pH 9.0). Unless otherwise stated, the standard reaction system consisted of 1 mL of buffer solution containing 1 μg/mL ZEN and either CaNF equivalent to 4 μg of immobilized ZENG or 4 μg of free ZENG. The reaction was allowed to proceed for 20 min, and unless otherwise specified, the temperature was maintained at 37 °C. After incubation, the reaction mixture was centrifuged, and the residual ZEN in the supernatant was determined by HPLC.

### 5.5. Degradation of ZEN in Food Matrices Using CaNF

ZEN was spiked into corn juice, wort, and beer to a final concentration of 1000 μg/kg. CaNF was then added at an equivalent immobilized ZENG concentration of 4 μg/mL, and the reaction mixture was incubated for 20 min. After the reaction, 1 mL of methanol was added to quench the reaction, and the sample was mixed for 2 min. The mixture was then centrifuged at 12,000 rpm for 10 min at 4 °C. The supernatant was collected, filtered through a 0.22 μm membrane, and transferred to a 2 mL HPLC vial for analysis.

For corn steep liquor, ZEN was spiked to a final concentration of 800 μg/kg and mixed thoroughly. CaNF was then added at an equivalent immobilized ZENG concentration of 4 μg/mL, and the degradation reaction was carried out at 30 °C and 350 rpm for 20 min. After the reaction, 1 mL of the treated sample was transferred into a 15 mL centrifuge tube, followed by the addition of 5 mL of 80% aqueous methanol. The mixture was vortexed for 3 min and sonicated for 15 min, and then centrifuged at 8000 rpm for 5 min at 4 °C. The supernatant was collected for further purification.

A C18 solid-phase extraction (SPE) cartridge (CNWBOND HC-C18, ANPEL Laboratory Technologies Inc., Shanghai, China) was conditioned with 5 mL of methanol and equilibrated with 5 mL of ultrapure water. The crude extract was then loaded onto the cartridge at a flow rate of 1–2 drops/s. After sample loading, the cartridge was rinsed with 3 mL of ultrapure water, and ZEN was eluted with 3 mL of methanol. The eluate was evaporated to dryness under a gentle stream of nitrogen at 50 °C, reconstituted in 1 mL of methanol, filtered through a 0.22 μm membrane, and subjected to HPLC analysis.

Detailed HPLC method evaluation for ZEN preliminary quantification in these matrices, including spike-recovery, precision, linearity, and LOD/LOQ, is provided in Section 5.8.

### 5.6. Preparation and Verification of Inactivated CaNF

Inactivated CaNF was prepared from the same batch of CaNF used for the evaluation of catalytic performances. Briefly, CaNF was heated in a water bath at 95 °C for 30 min to denature the enzyme component, followed by cooling to room temperature. The treated CaNF was collected by centrifugation, washed with deionized water for three times, and resuspended to the same concentration as the active CaNF. The loss of enzymatic activity was measured under the same reaction conditions used for the ZEN transformation assay. The residual activity was 2.5% of that of active CaNF, confirming effective inactivation. The inactivated CaNF was expected to preserve the matrix and relevant adsorption properties of CaNF. Therefore, it was used as a control material to distinguish the enzymatic activity of CaNF from non-enzymatic adsorption or matrix effects.

### 5.7. Determination of ZEN Concentration

ZEN concentrations were determined using an Agilent 1260 high-performance liquid chromatography (HPLC) system (Agilent Technologies, Santa Clara, CA, USA) equipped with a fluorescence detector and an Agilent C18 column (250 mm × 4.6 mm, 5 μm). The mobile phase consisted of methanol and water (80:20, *v*/*v*) at a flow rate of 0.6 mL/min. The column temperature was maintained at 30 °C, and the injection volume was 20 μL. Fluorescence detection was performed at an excitation wavelength of 274 nm and an emission wavelength of 440 nm. The degradation rate of ZEN was calculated using Equation (2):

Degradation rate (%) = (C_0_ − C_1_)/C_0_ × 100% (2)

where C_0_ and C_1_ represent the concentrations of ZEN in the solution before and after the reaction, respectively.

### 5.8. Preliminary Evaluation of the HPLC Method for ZEN Determination in Selected Food Matrices

The analytical performance of the HPLC method for ZEN determination was preliminarily evaluated in selected food matrices, including corn juice, wort, beer, and corn steep liquor. The evaluation included solvent-based calibration, LOD, LOQ, repeatability, and single-level spike-recovery experiments. Matrix-matched calibration, matrix effects, independent extraction efficiency, dilution integrity, and multi-level recovery were not systematically assessed in the present study.

#### 5.8.1. Calibration Curve and Linearity

Standard solutions of ZEN at different concentrations were prepared in methanol/water (50:50, *v*/*v*) and analyzed under the optimized HPLC conditions. Quantification was performed using a solvent-based external standard method. Calibration curves were constructed by plotting peak area against ZEN concentration, and linear regression analysis was used to evaluate linearity over the tested range. The linear range, regression equation, and correlation coefficient (R^2^) were recorded. Matrix-matched calibration was not included in the present study.

#### 5.8.2. LOD and LOQ

The limit of detection (LOD) and limit of quantification (LOQ) were determined using serially diluted ZEN standard solutions under the same chromatographic conditions. The LOD and LOQ were defined as the concentrations corresponding to signal-to-noise ratios of 3 and 10, respectively.

#### 5.8.3. Repeatability

Repeatability was evaluated by replicate analyses of ZEN-spiked matrix samples and expressed as the relative standard deviation (RSD, %).

#### 5.8.4. Spike-Recovery in Food Matrices

Single-level spike-recovery experiments were performed in corn juice, wort, beer, and corn steep liquor. Blank matrix samples were spiked with ZEN at 1000 μg/kg and processed according to the corresponding sample preparation procedures used for each matrix. ZEN concentrations were calculated using the solvent-based external calibration curve. Recovery was calculated as follows: (measured concentration/spiked concentration) × 100, and the results were expressed as mean ± SD and RSD (%).

### 5.9. Purification and Quantification of HZEN

ZEN was used as the substrate for enzymatic preparation of its hydrolyzed product, HZEN. Briefly, 10.00 ± 0.01 mg of ZEN (purity > 99%, powder) was dissolved in 5 mL of acetonitrile/water (10:90, *v*/*v*) and transferred to a 10 mL brown glass vial. 400 mg of CaNF was then added and the mixture was incubated at 35 °C with shaking at 350 rpm for 12 h.

After the reaction, an equal volume of methanol was added to terminate the enzymatic reaction. The mixture was centrifuged and the supernatant was filtered through a 0.22 μm nylon microfiltration membrane before preparative HPLC separation. The transformation product HZEN was collected using preparative HPLC according to a previously reported method with minor modification [40]. Briefly, the filtered reaction solution was injected into an Agilent 1100 HPLC system equipped with a Luna RP-C18 (2) semi-preparative column (250 × 10 mm, 5 μm) and a C18 security guard semi-preparative cartridge (10 × 10 mm). The column temperature was maintained at 25 °C, the injection volume was 500 μL, and the flow rate was 5.6 mL/min. Mobile phase A consisted of 80% ultrapure water, 20% acetonitrile, and 0.1% formic acid, while mobile phase B consisted of 100% acetonitrile and 0.1% formic acid. The gradient program was as follows: 0–3.5 min, 15% B; 3.5–33.5 min, 15–100% B; 33.5–34.5 min, 100% B; 34.5–35.0 min, 100–15% B; and 35.0–40.0 min, 15% B for column re-equilibration. UV detection was performed at 270 nm.

The acetonitrile and methanol in the collected HZEN-containing fraction obtained by preparative HPLC was removed by rotary evaporation at 45 °C. The remaining aqueous solution was further dried using a vacuum freeze-dryer at −40 °C and 10 Pa for 24 h to obtain the purified HZEN. The dried HZEN powder was accurately weighed using a high-precision electronic balance, reconstituted in methanol/water (50:50, *v*/*v*), and serially diluted to prepare standard solutions within the calibration range. The calibration curve was constructed by plotting peak area against HZEN concentration. After enzymatic conversion, the formed HZEN was quantified by HPLC using the HZEN calibration curve, whereas the remaining ZEN was quantified using the previously established ZEN calibration curve. The calibration curve of HZEN is shown in Figure S7. Column: Agilent ZORBAX Eclipse Plus C18 (4.6 mm × 250 mm, 5 μm). The mobile phase consisted of 0.1% (*v*/*v*) formic acid in water (A) and acetonitrile (B), with the following gradient program: 0–1 min, 50% B; 1–7 min, 50–80% B; 7–8 min, 80–50% B, followed by 1 min post-run equilibration. The column temperature was set at 35 °C, the flow rate was 0.8 mL/min, the injection volume was 20 μL, and the detection wavelength was set at 270 nm.

### 5.10. Characterization

The morphology of the samples was observed by scanning electron microscopy (SEM) using a Hitachi instrument (Hitachi High-Tech Corporation, Tokyo, Japan) at an accelerating voltage of 5.0 kV. Transmission electron microscopy (TEM) was performed on a JEOL JEM-2100F instrument (JEOL Ltd., Tokyo, Japan) at an accelerating voltage of 120 kV. High-angle annular dark-field scanning transmission electron microscopy (HAADF-STEM) and energy-dispersive X-ray spectroscopy (EDS) mapping were carried out on a JEOL JEM-2100F instrument operated at 200 kV. X-ray diffraction (XRD) patterns were recorded on an X-ray diffractometer (D8 ADVANCE, Bruker, Bremen, Germany) over a 2θ range of 20–50° at a scan rate of 5°/min. X-ray photoelectron spectroscopy (XPS) survey and high-resolution spectra were collected using an X-ray photoelectron spectrometer (Thermo Fisher Scientific, Shanghai, China). Fourier transform infrared (FT-IR) spectra were recorded on an FT-IR spectrometer (Nicolet iS50, Thermo Fisher Scientific, Waltham, MA, USA) over the wavenumber range of 500–4000 cm^−1^ using the KBr pellet method. Confocal laser scanning microscopy images were obtained using a Leica TCS SP8 instrument (Leica Microsystems, Wetzlar, Hessen, Germany). Thermogravimetric analysis (TGA) was carried out on a thermogravimetric analyzer (TGA 4000, PerkinElmer, Shelton, CT, USA) from 30 °C to 900 °C at a heating rate of 10 °C/min. The concentrations of Ca and P in liquid samples were determined by inductively coupled plasma atomic emission spectrometry (ICP-AES).

### 5.11. UPLC-Q-TOF/MS Analysis

The transformation product of ZEN was qualitatively analyzed by ultrahigh-performance liquid chromatography coupled with quadrupole time-of-flight mass spectrometry (UPLC-Q-TOF/MS). The system consisted of an Agilent 1290 ultrahigh-performance liquid chromatograph (Agilent Technologies, Santa Clara, CA, USA) equipped with a diode array detector and coupled to an Agilent G6538 accurate-mass quadrupole time-of-flight mass spectrometer with an electrospray ionization (ESI) source. Chromatographic separation was performed on an Agilent InfinityLab Poroshell 120 EC-C18 column (3.0 × 100 mm, 2.7 μm; Agilent Technologies, USA). The column temperature was maintained at 30 °C, the flow rate was 0.3 mL/min, and the injection volume was 2 μL.

The mobile phase consisted of water (A) and methanol (B). The gradient elution program was as follows: 0–10 min, 50% B; 10–20 min, 50–80% B; 20–30 min, 80% B; 30–31 min, 80–50% B; and 31–32 min, 50% B.

Mass spectrometric analysis was performed in negative ionization mode over an m/z range of 50–1000. The drying gas temperature was 300 °C, and the drying gas flow rate was 10 L/min. The capillary voltage, fragmentor voltage, and skimmer voltage were set at 3.5 kV, 125 V, and 50 V, respectively. MS/MS spectra were acquired at collision energies ranging from 15 to 35 eV. Data were processed using MassHunter Qualitative Analysis software (version B.06.00, Agilent Technologies, Santa Clara, CA, USA).

### 5.12. Cytotoxicity Assay of ZEN and Its CaNF-Catalyzed Transformation Product in HepG2 Cells

The cytotoxicity of ZEN and its transformation product HZEN was evaluated in HepG2 cells using a Cell Counting Kit-8 (CCK-8) assay. HZEN was collected by preparative HPLC, quantified by analytical HPLC. The residual ZEN was analyzed using HPLC. Residual ZEN was not detected in the reconstituted HZEN solution used for the HepG2 assay, with the signal below the LOD of the analytical method, indicating that ZEN contamination in the HZEN preparation was negligible. HepG2 cells were cultured in Dulbecco’s Modified Eagle Medium (DMEM) supplemented with 10% fetal bovine serum (FBS), 100 U/mL penicillin, and 100 μg/mL streptomycin. The cells were maintained at 37 °C in a humidified incubator with 5% CO_2_. The culture medium was renewed every 2–3 days, and the cells were passaged once or twice per week.

For the cytotoxicity assay, HepG2 cells were seeded into 96-well plates at a density of 2 × 10^4^ cells/well in 100 μL of complete medium and allowed to attach for 12 h. The medium was then replaced with fresh medium containing ZEN or HZEN at different concentrations (0, 2, 4, 6, 8, and 10 μg/mL), and the cells were incubated for different exposure times (0, 12, 24, 36, and 48 h). After treatment, 10 μL of CCK-8 reagent (Solarbio Science & Technology Co., Ltd., Beijing, China) was added directly to each well, and the plates were incubated at 37 °C in a 5% CO_2_ incubator for 2 h. The absorbance at 450 nm was measured using a microplate reader (Multiskan, Thermo Fisher Scientific, Waltham, MA, USA). Cell viability was calculated according to Equation (3). Each treatment was performed in triplicate, with three parallel wells for each concentration.

Cell viability (%) = (A_t_ − A_0_)/(A_c_ − A_0_) × 100% (3)

where A_t_, A_0_ and A_c_ represented the absorbance of the treated group, blank group and untreated control group, respectively.

### 5.13. COMSOL Simulation of the Protective Behavior of Porous Hydroxyapatite Under Acidic Conditions

COMSOL Multiphysics software (version 5.6, COMSOL AB, Stockholm, Sweden) was used to simulate the diffusion of hydrogen ions into porous hydroxyapatite (HAP) particles and the subsequent surface dissolution reaction under acidic conditions. The simulation was performed by coupling the Transport of Diluted Species (tds) and Surface Chemistry (sr) modules in a time-dependent study. The purpose of the simulation was to evaluate the potential protective effect of porous HAP against acid attack by characterizing hydrogen ion transport and HAP dissolution behavior.

The dissolution of hydroxyapatite was described by the following reaction:

Ca_10_(PO_4_)_6_(OH)_2_ + 8H^+^ → 10Ca^2+^ + 6HPO_4_^2−^ + 2H_2_O

Hydrogen ion transport in the aqueous phase was assumed to occur by free diffusion only, without convection. The external acidic environment was set to pH 5.0, corresponding to an H^+^ concentration of 0.01 mol/m^3^.

A two-dimensional geometric model was constructed consisting of a square aqueous domain with a side length of 6 μm and a porous spherical HAP particle with a diameter of 1 μm positioned at the center of the domain. The entire square domain, including the pore regions inside the particle, was defined as the aqueous phase to allow unrestricted hydrogen ion diffusion. The internal pore-wall surfaces of the porous HAP particle were defined as reactive boundaries where the dissolution reaction took place.

For the Transport of Diluted Species module, hydrogen ions were defined as the diffusing species throughout the aqueous domain. A constant hydrogen ion concentration of 0.01 mol/m^3^ was applied at the outer boundary to represent a stable acidic environment. The reactive pore-wall surfaces of HAP were coupled to the surface reaction and served as hydrogen ion consumption boundaries.

For the Surface Chemistry module, all pore-wall surfaces of the porous HAP particle were specified as reactive interfaces for hydroxyapatite dissolution. A physics-controlled mesh was used, with global mesh settings of normal/fine and local refinement applied to the reactive surfaces. The minimum mesh element size near the reactive boundaries ranged from 0.01 to 0.05 μm, and the mesh quality was maintained above 0.3.

A time-dependent solver was used for transient simulation over a period of 0–40 min with automatic time stepping enabled.

## Figures and Tables

**Table 1:** Comparison of kinetic parameters (Km and Kcat) of immobilized and free ZENG.

Catalyst	Km (μM)	Kcat (s^−1^)	Kcat/Km (s^−1^·μM^−1^)
CaNF	3.95	0.124	0.031
ZENG	8.21	0.051	0.006

Note: Km and Kcat values were calculated by fitting the initial reaction rates to the Michaelis–Menten equation. The corresponding Michaelis–Menten curves are shown in Figure S4.

**Table 2:** Matrix spike recoveries of ZEN in different matrices.

Matrix	Spiked Level (μg/kg)	Measured (μg/kg, mean ± SD)	Recovery (%)	RSD (%)
corn juice	1000	879.77 ± 31.15	87.98	3.54
wort	1000	976.31 ± 2.25	97.63	0.23
beer	1000	945.55 ± 22.01	94.56	2.32
corn steep liquor	1000	892.12 ± 14.11	89.21	1.58

Values are expressed as mean ± SD (n = 3). Recovery (%) was calculated as (mean measured concentration/spiked concentration) × 100. RSD indicates relative standard deviation.

## Data Availability

The original contributions presented in this study are included in the article/Supplementary Material. Further inquiries can be directed to the corresponding author.

## Supplementary Materials

The following supporting information can be downloaded at https://www.mdpi.com/article/10.3390/toxins18050229/s1: Figure S1. FT-IR spectra of (A) CaNF-pH 6.5 and (B) CaNF-pH 8.0. Figure S2. Immobilization efficiency of ZENG at different initial enzyme concentrations. Figure S3. Relative activity of CaNF (pH 8.0) at different temperatures. Figure S4. Michaelis–Menten kinetics of (A) immobilized enzyme (CaNF) and (B) free enzyme (ZENG), showing the relationship between substrate concentration and reaction velocity. Figure S5. Linear correlation between peak area and ZEN concentration in the range of 100–1000 ng/mL. Figure S6. HPLC chromatogram of ZEN at different concentrations. Figure S7. HPLC calibration curve for HZEN. Figure S8. The mass spectra of the ZEN dissociation products analyzed by UPLC-Q-TOF/MS. Figure S9. The mass spectra of the HZEN dissociation products analyzed by UPLC-Q-TOF/MS. Figure S10. Possible dissociation pathway of ZEN. Table S1. The molar ratio of Ca to P in CaNF-pH 8.0. Table S2. Enzyme loading content of CaNF and normalization of nominal ZENG amount used in comparative catalytic assays. Table S3. Determination of LOD and LOQ for ZEN by HPLC. Table S4. Molar balance between ZEN consumption and HZEN formation during enzymatic conversion. Table S5. Ecotoxicological Data of ZEN and HZEN Predicted by U.S. EPA ECOSAR and T.E.S.T. Software.
