# Supplementary material for: Efficient Biotransformation of Zearalenone in Acidic Food Matrices by Alkaline Enzyme–Inorganic Hybrid Nanoflower
Source: Toxins (Basel). 2026 May 13;18(5):229. doi: 10.3390/toxins18050229 (PMC13211546; doi:10.3390/toxins18050229)
Supplement: Supplementary file 1 [file toxins-18-00229-s001.zip › toxins-4177386-supplementary.pdf]

Supplementary materials: Biotransformation of Zearalenone in Acidic Food Matrices by an Alkaline Enzyme-Inorganic Hybrid nanoflower

Ping Ding, Wenchao Liao, Chenyu Chen, Xincheng Chen, Chengfei Wang, and Xiaoyang Li

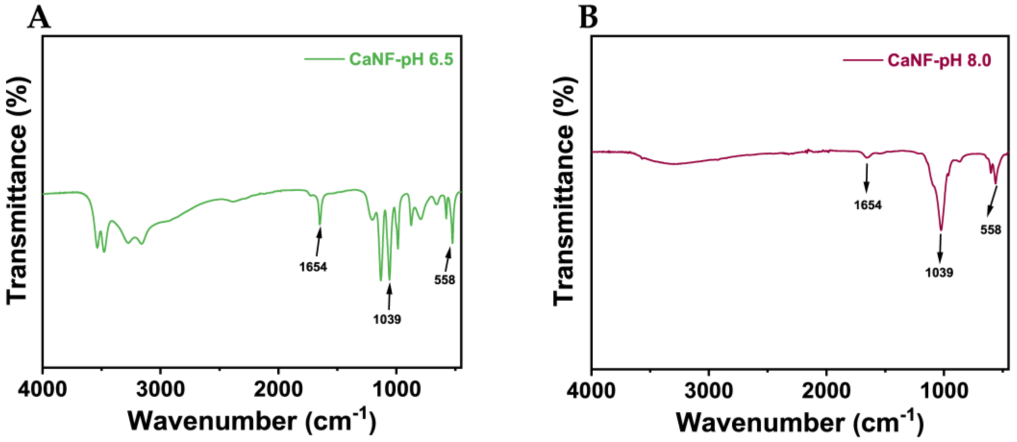

Figure S1. FT-IR spectra of (A) CaNF-pH 6.5 and (B) CaNF-pH 8.0.

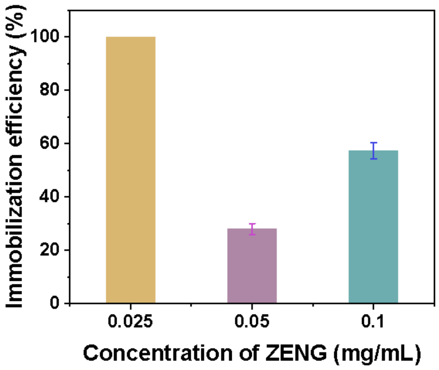

Figure S2. Immobilization efficiency of ZENG at different initial enzyme concentrations.

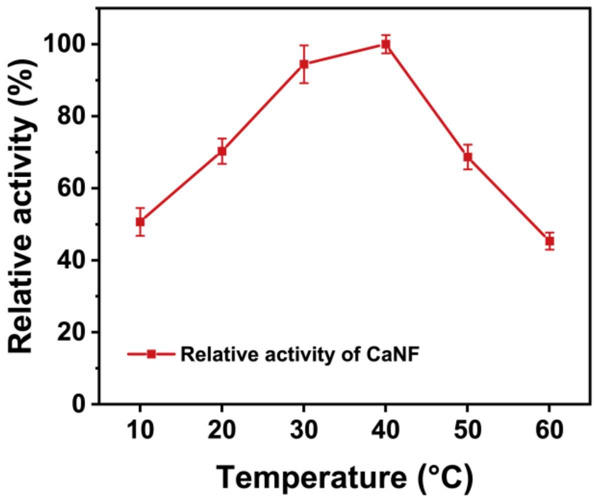

Figure S3. Relative activity of CaNF-pH 8.0 at different temperatures.

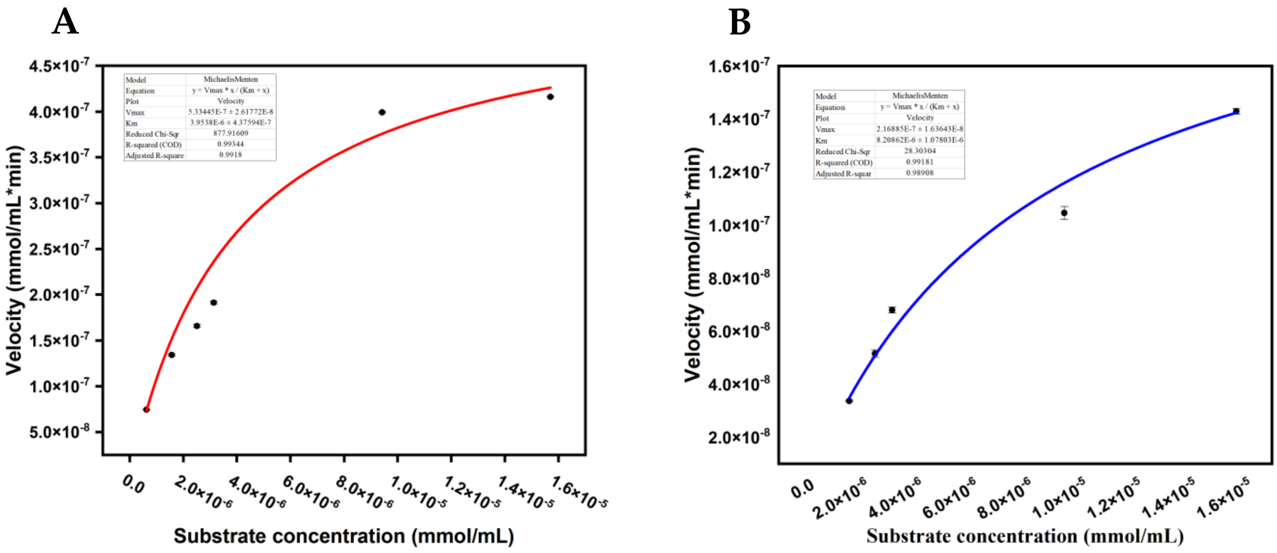

Figure S4. Michaelis–Menten kinetics of (A) immobilized enzyme (CaNF) and (B) free enzyme (ZENG), showing the relationship between substrate concentration and reaction velocity.

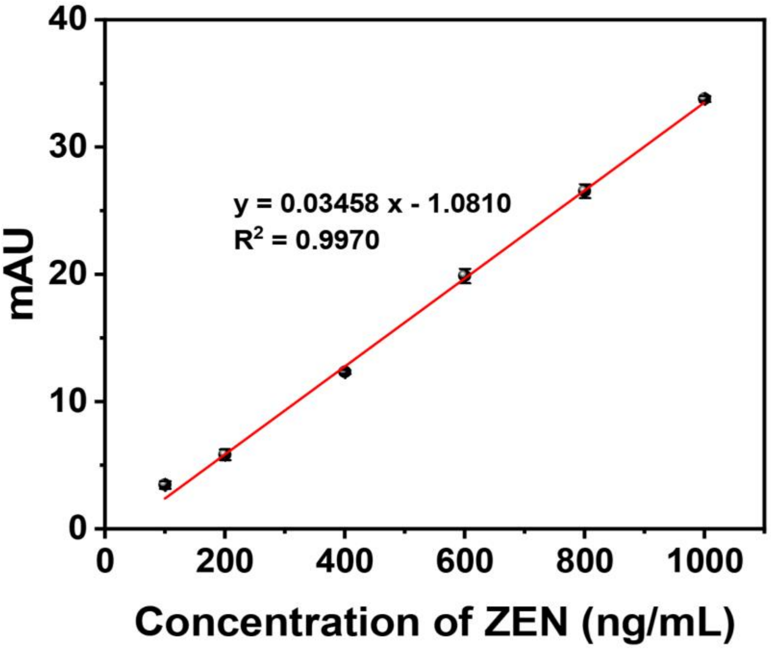

Figure S5. Linear correlation between peak area and ZEN concentration in the range of 100–1000 ng/mL.

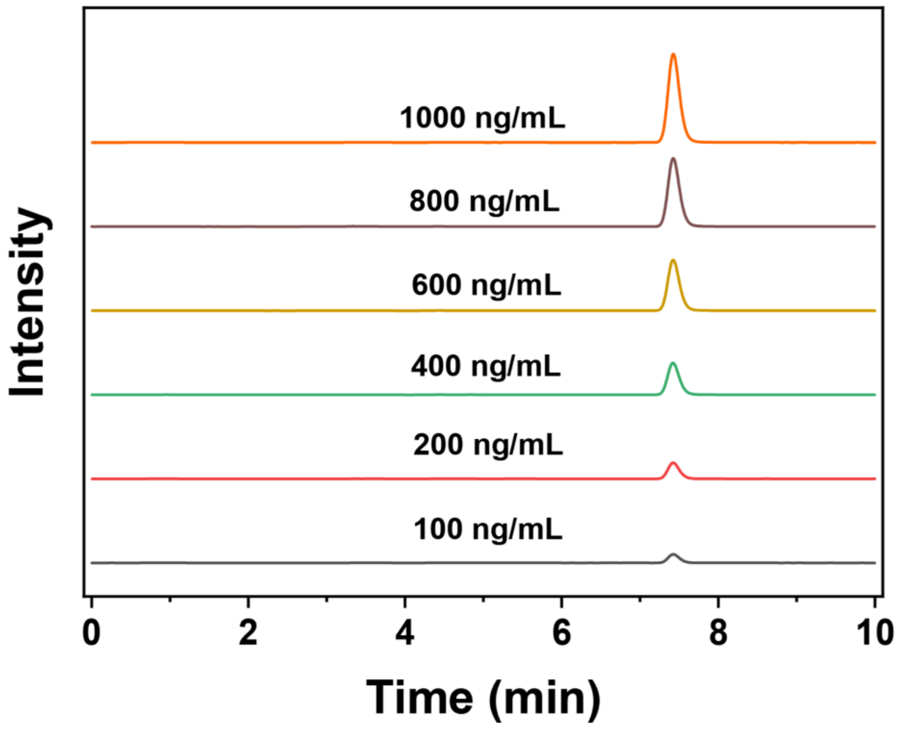

Figure S6. HPLC chromatograms of ZEN at different concentrations.

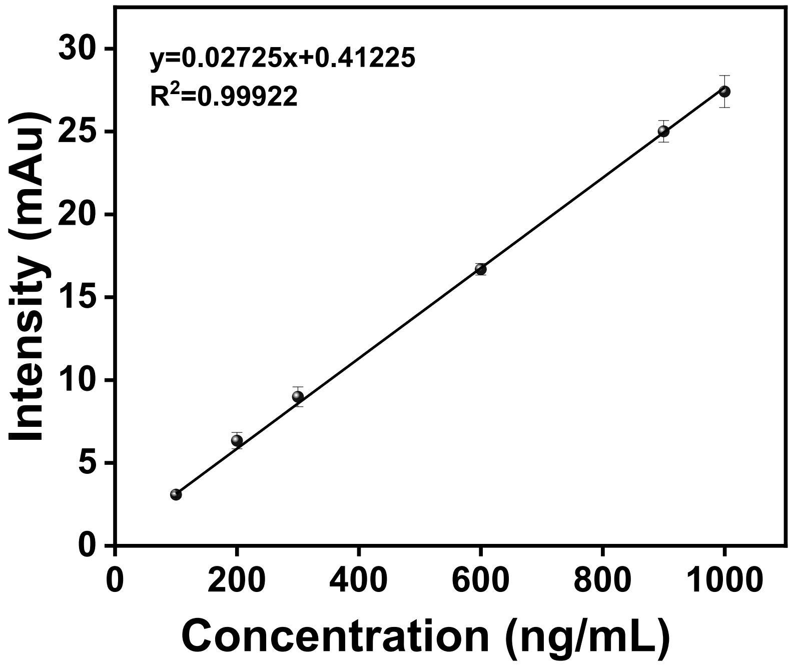

Figure S7. HPLC calibration curve for HZEN.

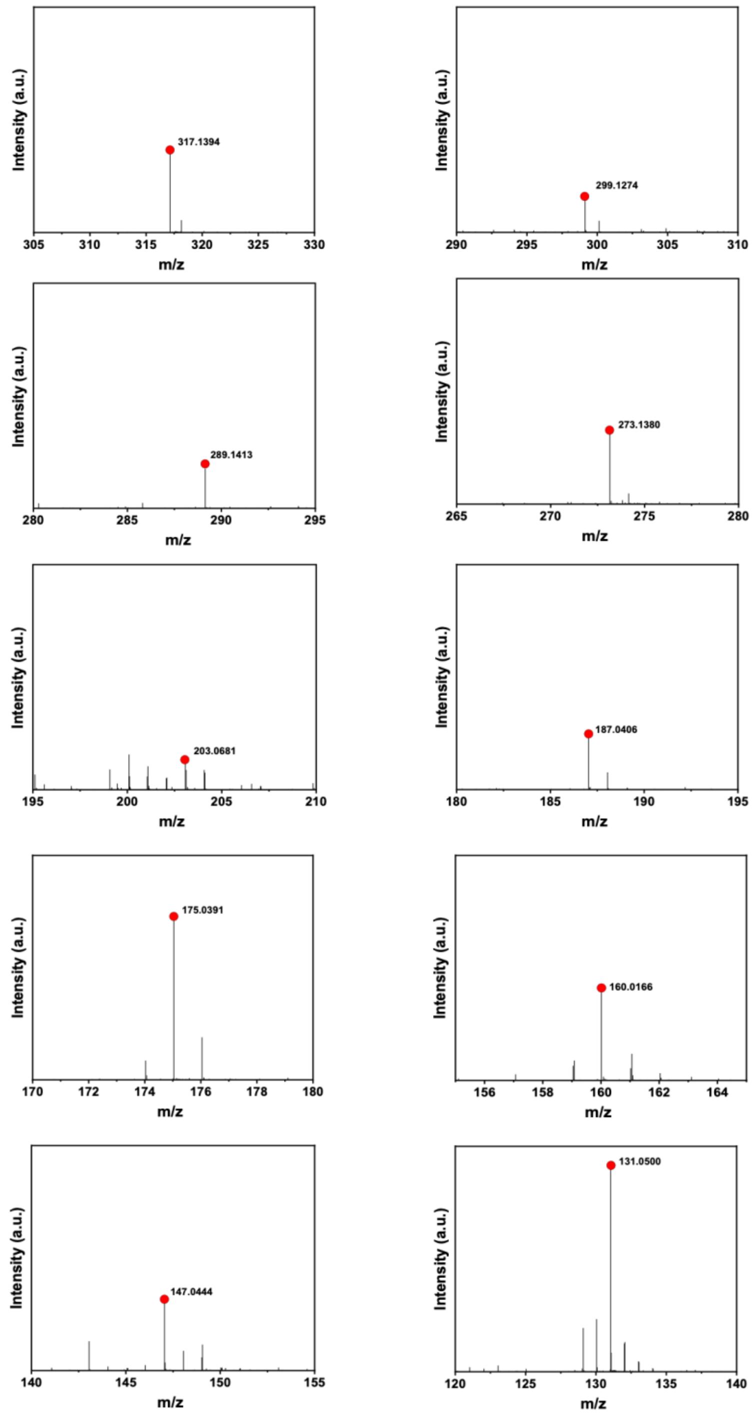

Figure S8. The mass spectra of the ZEN dissociation products analyzed by UPLC-Q-TOF/MS.

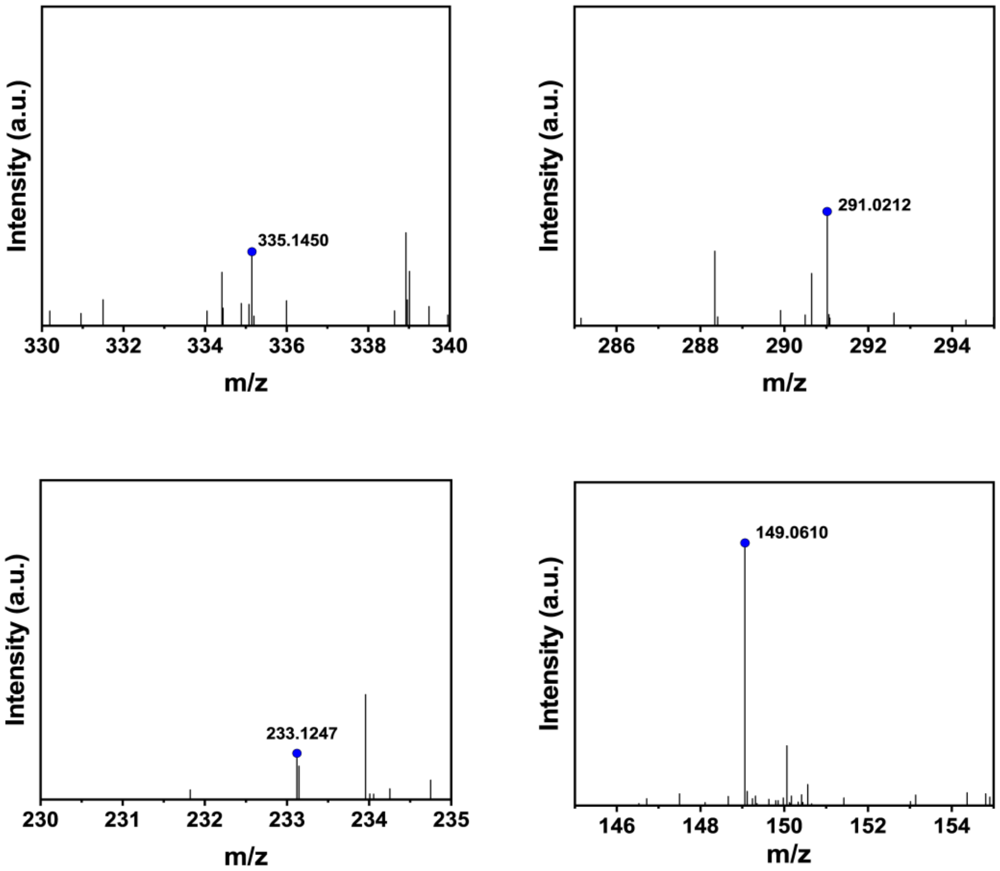

Figure S9. The mass spectra of the HZEN dissociation products analyzed by UPLC-Q-TOF/MS.

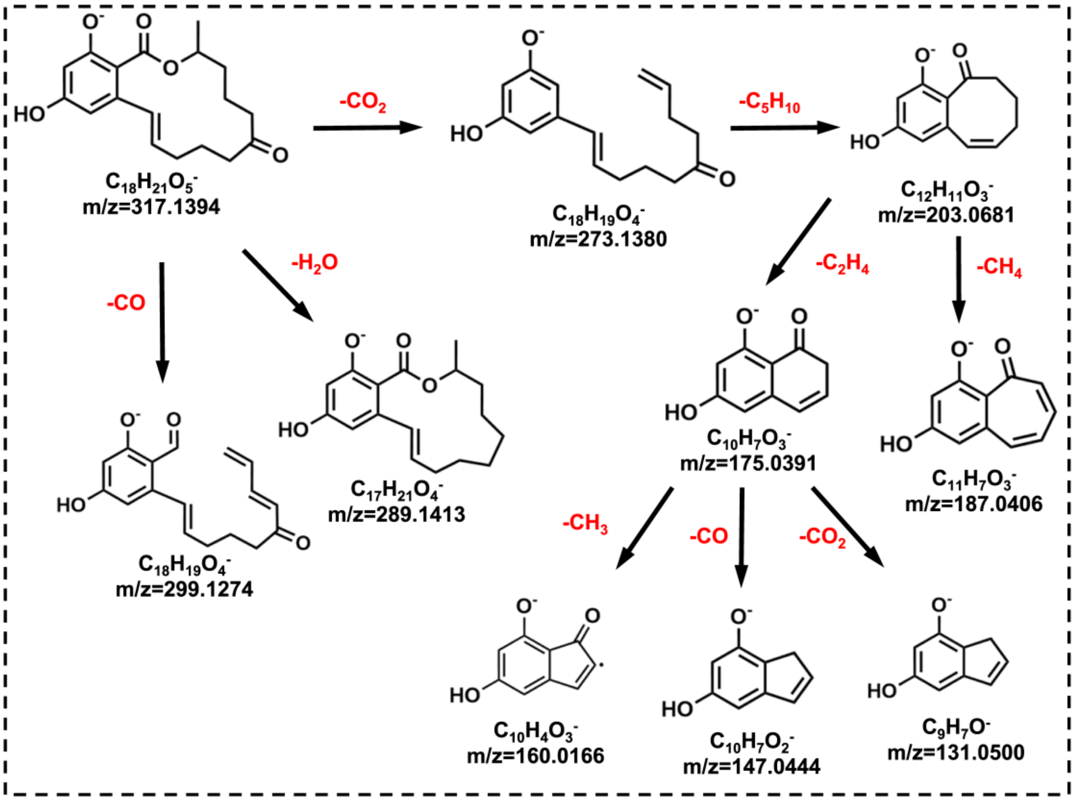

Figure S10. Possible dissociation pathway of ZEN.

Table S1. The molar ratio of Ca to P in CaNF-pH 8.0.

Sample	Ca/P
CaNF-pH 8.0	1.6

Table S2. Enzyme loading content of CaNF and normalization of nominal ZENG amount used in comparative catalytic assays.

Sample	Total materials used in assay (μg)	Enzyme loading content (%)	Nominal ZENG amount in assay (μg)
CaNF-pH 8.0	40	10.0	4.0
CaNF-pH 6.5	49	8.1	4.0
CaNF-pH 9.0	70	5.7	4.0
free ZENG	4	N/A	4.0

Enzyme loading content was defined as the mass percentage of immobilized ZENG in the final CaNF material. The amount of CaNF used in comparative catalytic assays was calculated according to the BCA-determined enzyme loading content, and the same nominal amount of free ZENG was used in the corresponding control experiments.

Table S3. Determination of LOD and LOQ for ZEN by HPLC.

**LOD (ng/mL)**	0.82
**LOQ (ng/mL)**	2.73

Table S4. Molar balance between ZEN consumption and HZEN formation during enzymatic conversion.

**Sample No.**	Initial ZEN (μM)	Remaining ZEN (μM)	ZEN consumed (μM)	HZEN formed (μM)	HZEN yield (% of consumed ZEN)	Overall molar recovery (%)
**1**	10.00	0.82	9.18	8.76	95.42	95.80
**2**	10.00	0.63	9.37	9.05	96.58	96.80
**3**	10.00	0.56	9.44	9.17	97.17	97.33
**Mean ± SD**	10.00 ± 0	0.67 ± 0.13	9.33 ± 0.13	8.99 ± 0.21	96.39 ± 0.89	96.64 ± 0.78

Data represent three independent replicate experiments (n = 3). ZEN consumed = Initial ZEN − Remaining ZEN. HZEN yield (% of consumed ZEN) = HZEN formed / ZEN consumed × 100%. Overall molar recovery (%) = (Remaining ZEN + HZEN formed) / Initial ZEN × 100%..

Table S5. Ecotoxicological data of ZEN and HZEN predicted using U.S. EPA ECOSAR and T.E.S.T. software.

**compound**			ZEN	HZEN
ECOSAR	acute toxicity (mg/L)[a]	Fish	4.26	702.47217
		Daphnid	8.91	424.021
		Green Algae	3.29	406.72104
	chronic toxicity (mg/L)[b]	Fish	3.33	73.799904
		Daphnid	1.63	49.02301
		Green Algae	1.17	122.05061
**T.E.S.T**	fathead minnow[c]	predicted value	0.35	1.59
	developmental toxicity	predicted value	0.80	0.54
	mutagenicity	predicted value	0.05	0.04
	bioaccumulation factor	predicted value	4.84	1.07
	Daphnia magna[c]	predicted value	11.21	49.96
	rat oral[c]	predicted value	466.23	829.66

[a]Acute toxicity (mg/L): 96 h LC_{50} for fish, 48 h LC_{50} for Daphnia, and 96 h LC_{50} for green algae. [b]Chronic toxicity (mg/L): predicted chronic toxicity values based on model outputs (e.g., NOEC or EC_{20}, depending on the software). [c]T.E.S.T. predictions: 96 h LC_{50} (mg/L) for Fathead Minnow, 48 h EC_{50} (mg/L) for Daphnia magna, and LD_{50} (mg/kg) for rat oral toxicity.
